# Validation of the Employment Precariousness Scale and its associations with mental health outcomes: results from a prospective community-based study of pregnant women and their partners in Dresden, Germany

**DOI:** 10.1136/bmjopen-2023-077206

**Published:** 2024-08-30

**Authors:** Marlene Karl, Andreas Staudt, Alejandra Vives, Marie Kopp, Victoria Weise, Judith T Mack, Susann Steudte-Schmiedgen, Andreas Seidler, Susan Garthus-Niegel

**Affiliations:** 1 Department of Psychotherapy and Psychosomatic Medicine, Faculty of Medicine, Technische Universität Dresden, Dresden, Germany; 2 Institute and Policlinic of Occupational and Social Medicine, Faculty of Medicine, Technische Universität Dresden, Dresden, Germany; 3 Institute for Community Medicine, Section Methods in Community Medicine, University Medicine Greifswald, Greifswald, Germany; 4 Public Health, Pontificia Universidad Catolica de Chile, Santiago, Chile; 5 Institute for Systems Medicine (ISM) and Faculty of Medicine, MSH Medical School Hamburg University of Applied Sciences and Medical University, Hamburg, Germany; 6 Department of Childhood and Families, Norwegian Institute of Public Health, Oslo, Norway

**Keywords:** mental health, postpartum period, postpartum women, public health, psychometrics

## Abstract

**Abstract:**

**Objective:**

To translate the Employment Precariousness Scale (EPRES) from Spanish into German (EPRES-Ge), adapt it to the German context, assess the psychometric properties and show prospective associations with mental health outcomes within the peripartum period.

**Design:**

Analyses encompassed descriptive statistics, exploratory factor analysis, confirmatory factor analysis (CFA) to validate the structure of the EPRES, and multivariate regression analyses with mental health outcomes 8 weeks after birth.

**Participants:**

Self-report data from 3,455 pregnant women and their partners within the Dresden Study on Parenting, Work, and Mental Health prospective longitudinal cohort study were used.

**Results:**

The EPRES-Ge with five dimensions and 20 items showed good internal consistency (Cronbach’s α=0.77). All scales showed good reliability coefficients of α=0.73–0.85 and good item-subscale correlations of r=0.63–0.98, with the exception of subscale rights, which showed poor reliability of α=0.30 and item-subscale correlations of r=0.45–0.68. Exploratory analysis and CFA confirmed the proposed five-dimensional structure, explaining 45.08% of the cumulative variance. Regression analyses with mental health outcomes after birth revealed statistically significant associations (*β=*0.12–0.20).

**Conclusions:**

The EPRES-Ge is a valuable tool for assessing employment precariousness as a multidimensional construct. The scales could be adapted to the German working context. Precarious employment, as measured by the EPRES-Ge, is a determinant of mental health problems in young families.

**STRENGTHS AND LIMITATIONS OF THIS STUDY**

- Using exploratory factor analyses in SPSS and confirmatory factor analyses in Mplus, this study translated, validated and adapted the Employment Precariousness Scale, a multidimensional instrument for assessing precarious employment, in a German sample.
- The validation was done in a large prospective-longitudinal cohort study with a high sample size of 3,455 pregnant women and their partners within the region of Dresden, Germany.
- While the selective sample consisting of relatively young women and men expecting a baby provides valuable insights into the peripartum period, it is a limitation in terms of the generalisability of the results to other populations.

## Introduction

 Employment in Germany has undergone massive changes in recent decades due to technological innovation, globalisation and more flexible working arrangements. Companies are pursuing a strategy of more flexible employment^1^ to better adapt to market requirements and ultimately relax what were considered to be strict employment regulations. This has been supported by the German government introducing new structures (the ‘Hartz Reforms’) within the turn of the millennium to increase the flexibility of the labour market,^2 3^ which has increased in part-time, marginal and temporary employment; working poverty; and minimum wage,^4 5^ representing an increase in precarious employment (PE) and a decline of standard employment conditions.^6^ PE itself can be used as a broad term to describe forms of non-standard employment, although there is no universally accepted definition of PE.^7 8^

PE as a construct can be understood as a social determinant of poor health, affecting not only individuals but also families and society as a whole.^7 9^ As a result, research interest in this area has grown rapidly, leading to new insights into different aspects of the employment relationship and their impact on health. Previous research has often focused on unidimensional/individual aspects of PE, particularly job insecurity and temporary employment,^10 11^ and their association with health. For example, research in Germany has shown that insufficient income^12^ and job insecurity are associated with poor overall health,^13^ and low income and financial strain are associated with lower health and life satisfaction.^14^ It has also been hypothesised that PE affects not only general health but also mental health, acting as a stressor on individuals and predisposing them to mental health problems when exposed to it for a prolonged time.^9^ This association can be found in many European countries, including Germany, where poor self-rated (mental) health was linked to increasing PE.^15 16^ However, studying the effects can be quite complex due to various factors such as bidirectional causality, confounding variables and selection effects.^17^ In the case of temporary employment, for example, it seems to matter whether workers chose a temporary contract or whether it was the only contract they were offered. Only involuntary temporary employment was associated with negative mental health outcomes.^11^ Theoretical approaches are still lacking, but initial conceptual models link PE and health in the wider context of the political context and its impact on the labour market, together with PE consequences such as more hazardous working conditions and material deprivation.^6^

To better understand the underlying contributing mechanisms, recent systematic reviews and a meta-analysis concluded that multidimensional approaches, rather than unidimensional approaches such as insufficient income, yield stronger associations with mental health in terms of effect sizes.^8 11 18^ In addition, evidence suggests that PE is indeed associated with mental health problems such as psychological distress, depression, anxiety and mental health symptoms^8 18^; however, many longitudinal studies on PE and mental health showed limitations within the study design.^11^

In particular, women are more exposed to PE^19^,^21^ and psychosocial risk factors than men, resulting in a stronger association between PE and mental health in women in most cases.^22^ Although different causal pathways between women and men are being discussed in a recent literature review such as the male breadwinner model, where men are responsible to provide for the family while women are responsible for care work,^23^ new research suggests that not only are mothers at a disadvantage in the workplace but also the very fact that age and gender (a woman of childbearing age) result in more PE due to the anticipation of the women having children, and therefore additional costs to the organisation.^24^ As pregnancy is already a particularly vulnerable time for women, PE must also be considered in the context of peripartum health. It has been highlighted that the pregnancy period, with its many changes and influencing factors, can lead to a greater impact on the working conditions of female workers.^25^ While employment has been shown to generally have a positive effect on the mental health of mothers,^26 27^ some occupational risk factors and working conditions have already been associated with adverse pregnancy outcomes^28^,^32^ with persistent negative effects of PE during pregnancy for the mental health of the mother in the early postpartum period.^33 34^ However, research is still sparse on the effect of PE and mental health during the peripartum period.

### The EPRES

To close the previously mentioned gap of unidimensional approaches of PE, Amable *et al*^35^ developed and then Vives *et al*^36^ validated a multidimensional instrument assessing PE within the Research Group on Health Inequalities, Environment – Employment Conditions Network. The EPRES covers six dimensions: *temporariness* (employment instability), *disempowerment* (individual level bargaining over employment conditions), *vulnerability* (defenselessness to authoritarian treatment), *wages* (low or insufficient; possible economic deprivation), *rights* (lack of entitlement to, or knowledge about, workplace rights and social security benefits) and *exercise rights* (powerlessness to exercise workplace rights and social security benefits).

The scale was developed with the aim to contribute to the improvement of employee’s health through possible new work policies.^10^ The measure has already been validated within Spanish, Chilean, Catalonian, Greek and Swedish samples.^36^,^39^ The multidimensional approach of the EPRES was specifically devised for epidemiological studies among waged workers and has shown good acceptability, internal consistency (Cronbach’s alpha coefficients of ≥0.70) and construct validity.^36^ It was found to be strongly associations with occupational injuries than individual construct measures such as job insecurity and temporary employment.^10^ Moreover, the overall score was found to be linked to poor general and mental health^40^,^42^ and, in particular, to perceived stress in men and women.^43^ Our working group has already investigated the role of PE in the peripartum period using the EPRES in a German sample of pregnant women and their partners and has found a positive association of PE during pregnancy with postpartum depressive symptoms 8 weeks after birth after controlling for previous depressive symptoms.^33 34^ There is first evidence that the subscale *vulnerability* has the highest association with perceived general somatic and mental health.^36^ This association appears to follow a positive dose-response function, *ie*, the higher the PE, the higher the prevalence of poor mental health.^9 21 37 41 42^

Against this background, this study aimed to (a) translate the revised EPRES-2010^36^ from Spanish into German (EPRES-Ge), (b) adapt it to the German context, (c) assess the psychometric properties and (d) show prospective associations with mental health outcomes within the peripartum period, namely, symptoms of depression, somatisation, anxiety, obsessive-compulsiveness and anger/hostility, and further explore possible sex differences within this association.

## Methods

### Subjects and study design

This study is part of the longitudinal cohort ‘Dresden Study on Parenting, Work and Mental Health’ (**DREAM**: “**DR**esdner Studie zu **E**lternschaft, **A**rbeit und **M**entaler Gesundheit”), which prospectively examines the relationship between parental work participation, role distribution, stress factors and their effects on perinatal outcomes and long-term family mental and somatic health.^44^ Data collection and management were facilitated by the Research Electronic Data Capture, a secure, web-based application for data capture as part of research studies, hosted at the ‘Koordinierungszentrum für Klinische Studien at the Faculty of Medicine of the Technische Universitat Dresden.^45 46^ Expectant mothers and their partners were recruited within the wider city of Dresden, Germany. The measurement points encompass T1 (during pregnancy), T2 (8 weeks after birth) and four additional postpartum assessment waves (for a detailed description of the study see.^44^ For the purpose of the present study, self-report data from expectant mothers and their partners were used to validate the EPRES-Ge. At time of data extraction, n*=*2,209 expectant mothers and n*=*1592 partners had returned the first questionnaire (T1) containing the EPRES-Ge. Of these, n=2,001 expectant mothers and n=1,454 expectant partners fulfilled the inclusion criteria (self-reported) of working full-time, part-time, being in marginal employment, apprenticeship, or already on maternal leave or employment ban from a previous job at T1 (during pregnancy). Concerning T2 (8 weeks after birth), 1,734 mothers and 1,193 partners of the sample at T1 (*n* slightly varies between analyses due to missing data) had returned their T2 questionnaires. Participants with self-employment as main occupation were excluded.

### Translation and adaptations of the EPRES-Ge

The process of translating and adapting the Spanish version of the original EPRES and the revised EPRES-2010 to the EPRES-Ge consisted of four steps, and the final adaptations are outlined below:

#### Temporariness

In the original EPRES-2005, participants were asked at the beginning of the study whether they worked on a permanent or non-permanent contract. Participants who reported having a permanent contract were not asked about the duration of their contract and were given a temporariness score of 0 as an indicator of no PE for this section. While the suggested response categories for contract duration questions remained the same as EPRES-2005, a free text option was introduced for participants to specify contract durations. The research team later categorised the responses from the free text field within the provided response options.

#### Disempowerment

Due to the different economic system between Germany and Spain, uestions on disempowerment were changed from EPRES-2005. The original questions included information on whether working time, working hours,and wages were set by *collective agreement, by the employer* or *don't know*. In the German translation, these items were changed to questions on the type of employment contract and the involvement of parties such as trade unions, works councils and the German government (*national trade union agreement, company agreement, employment contract, don't know* and *others*). Because of this major adaptation and the different theoretical approach, the disempowerment scale was excluded from any further analysis (exploratory analysis and CFA) because of its reduced comparability with the original EPRES.

#### Vulnerability

The *vulnerability* scale was answered with five EPRES-2005 items on the original five-point ordinal scale.

#### Wages

The response categories for the EPRES-2005 item on the *ability of covering basic needs* were slightly reduced by one to *not at all, a little, a good amount* and *very much,* while the response categories of the item *ability to cover unexpected expenses* remained unchanged. The response categories of the item on monthly net salary were slightly adapted to the German labour market: (4) *up to 450€*, (3) *451–850€*, (2) *851–1,500€*, (1) *1,501–2,500€* and (0) *more than 2,500€*.

#### Rights

The items were taken from the revised EPRES-2010, with the exception of the wording of the question *pension due to old age and disability* was changed into *company pension plan,* as the German social security system covers general old age and disability pensions where applicable. The response categories for the remaining items (*pension, severance pay, parental leave or unemployment benefit*) were not altered (*yes, no or do not know*).

#### Exercise rights

The questions were derived from the revised EPRES-2010 and remained the same as in the original measure.

In summary, the final version of the EPRES-Ge consisted of 22 items and five dimensions (see online supplemental material 1). As in the original EPRES-2005 and EPRES-2010, the subscale scores were calculated as averages and transformed into a 0–4 scale. The global score is the average of five subscales, ranging from 0 to 4, where 0 represents the lowest and four the highest level of precariousness.^36 47^

### Mental health measures

To examine construct validity, we examined prospective associations with mental health outcomes measured at T2, approximately 8 weeks after birth. We analysed associations with symptoms of *somatisation, anxiety, obsessive-compulsiveness* and *hostility*, which were assessed using the validated German version of the Symptom-Checklist Revised (SCL-90-R;)^48^, a measure to assess symptomatic distress . The SCL-90-R is a questionnaire frequently used in clinical practice to assess psychological distress.^49^ The chosen scales ranged from 0 to 40. Higher scores indicate a greater symptom burden.^48^

Furthermore, symptoms of postpartum depression (PPD) were measured by the German version of the Edinburgh Postnatal Depression Scale (EPDS).^50 51^ The EPDS is the most common scale to screen for symptoms of PPD across the perinatal period and has been validated in numerous studies.^52^ It is a 10-item self-report measure, is scored on a four-point scale (0–3) and ranges from 0 to 30. Higher scores indicate increased levels of PPD. The EPDS includes feelings of anxiety, guilt or sadness, sleep problems or thoughts of harming oneself.

### Statistical analysis

Item and scale descriptive statistics were calculated, and listwise deletion was used for missing values. The factorial structure of the EPRES-Ge was investigated using exploratory analysis and CFA. For the purpose of cross-validation, two random subsamples were generated using a random number generator in Microsoft Excel. To explore the underlying factorial structure, principal axis factor analysis with varimax rotation was used in the first random subsample (n=1,718), extracting factors with eigenvalues of >1.

Next, the hypothesised factorial structure was then tested using CFA in the second random subsample (n=1,737). A single step, five-factor measurement model was applied, allowing the factors to correlate freely. Model fit was assessed using the comparative fit index (CFI) and the root mean square error of approximation (RMSEA). Cut-off values for both indices were set according to the literature at ≥0.90 for the CFI^53^ and at ≤0.05 for the RMSEA.^54^

To investigate prospective associations of PE and mental health outcomes, multivariate regression analyses with the EPRES-Ge score as the independent variable and respective mental health outcomes as dependent variables were conducted. Younger age, anxiety and low educational level might be a risk factor for PPD^55^ and might be associated with the perception of a less stable/favourable employment as well as the female sex, who typically work in more PE conditions.^56^ Therefore, we included the covariates age, sex (female/male) and education (university degree yes/no) within our analyses of PE and mental health outcomes. To further explore sex differences within the subscales, regression analyses were repeated separately for women and men. Analyses were performed using SPSS 27 and Mplus version 7.4.^57^

### Patient and public involvement statement

None.

## Results

### Study population and retention rate

The total sample consisted of 55 expectant mothers and their partners (table 1). The sample was well educated, with 55.0% reporting a high educational background, that is, a university degree. Participants were mostly employed on permanent contracts (80%), representing a sample with a relatively high standard of employment. Almost all participants (96.3%) reported living permanently with their partner, and 80.4% of participants reported that they were expecting their first child.

**Table 1:** Sample description of mothers and their partners at T1 (N = 3,455*)

Sample characteristics	Total (n=3,455)	
	n*(%)	M±SD (range)
Expectant mothers	2,001 (57.9)	
Pregnancy week		29.46±6.37 (8–42)
Partners	1,454 (42.1)	
Age		31.38±4.51 (20–56)
Country of birth		
Germany	3,320 (96.4)	
Other	123 (3.6)	
Education		
Below or 10 years	903 (26.2)	
More than 10 years	2,540 (73.8)	
Education		
No university degree	1,547 (45.0)	
University degree	1,887 (55.0)	
Employment status††		
Full-time employment	2,278 (65.9)	
Part-time employment	435 (12.6)	
Marginal employment	103 (3.0)	
Still in apprenticeship	35 (1.0)	
Employment ban	309 (8.9)	
Parental leave	623 (18.0)	
Monthly income of main job (after taxes)		
Less than 450 €	74 (2.2)	
451–850 €	62 (1.8)	
851–1500 €	697 (20.3)	
1501–2500 €	1,965 (57.4)	
More than 2,500 €	628 (18.3)	
Mental health outcomes at T2		
SCL-Somatisation‡	2,914	3.32 3.48 (0–34)
SCL-Anxiety‡	2,914	1.59±2.54 (0–29)
SCL-Obsessive-compulsive‡	2,913	3.80±3.84 (0–25)
SCL-Hostility‡	2,912	1.77±2.13 (0–20)
EPDS§	2,927	4.91±3.81 (0–25)

* n slightly varies due to missing data.

† Multiple answers allowed.

‡ Symptom-Checklist Revised.

§ Edinburgh Postnatal Depression Scale.

MmeanNsample size

### Item descriptive statistics

Table 2 shows means *M*, SD, proportion of missing values, response value frequencies and item-subscale correlations for each item. Overall, the response was high (97.0–99.0%) indicating good acceptability of the questionnaire. Almost all response options were used. No participant reported that their monthly income never covered their basic needs.

**Table 2:** Item-descriptive statistics of the sample of mothers and their partners at T1

Item	Missing	*M*	*SD*	Response value frequency (%)						Pearson item-subscale correlations^***^				
	(%)			0	1	2	3	4	T	D	V	W	R	ER
Temporariness														
Duration of contract	1.3	0.27	0.64	2,727 (80.0)	570 (16.7)	22 (0.6)	63 (1.8)	27 (0.8)	0.98**	−0.05**	0.007	0.17**	0.07**	−0.05*
Months under temporary contracts previous year	3.0	0.69	1.48	2,726 (81.3)	36 (1.1)	13 (0.4)	50 (1.5)	526 (15.7)	0.98**	−0.12**	0.022	0.06**	0.02	−0.03
Disempowerment														
Working hours settled	0.9	0.77	0.54	975 (28.5)	2249 (65.7)	200 (5.8)			−0.03	0.90**	−0.00	0.17**	0.18**	−0.01
Salary settled	1.0	0.67	0.57	1,295 (37.9)	1,946 (56.9)	180 (5.3)			−0.15**	0.91**	−0.01	0.13**	0.18**	−0.05**
Vulnerability														
Afraid to demand better working conditions	0.8	0.810	0.97	1,674 (48.8)	1,007 (29.4)	508 (14.8)	201 (5.9)	37 (1.1)	0.03	0.03	0.74**	0.19**	0.07**	0.33**
Defenseless towards unfair treatment	0.7	0.692	0.97	1,970 (57.4)	835 (24.3)	378 (11.0)	205 (6.0)	42 (1.2)	0.00	−0.00	0.81**	0.13**	0.04*	0.34**
Afraid of being fired for not doing…	0.9	0.394	0.74	2,471 (72.2)	658 (19.2)	213 (6.2)	62 (1.8)	20 (0.6)	0.08**	0.01	0.64**	0.13**	0.02	0.20**
Treated in authoritarian manner	0.87	1.077	1.11	1,288 (37.6)	1,167 (34.1)	497 (14.5)	366 (10.7)	107 (3.1)	−0.06**	−0.04*	0.70**	0.13**	0.03	0.30**
Made to feel easily replaceable	1.0	0.569	0.94	2,230 (65.2)	701 (20.5)	290 (8.5)	139 (4.1)	62 (1.8)	0.06**	−0.02	0.72**	0.15**	0.02	0.26**
Wages														
Cover basic needs?	0.6	0.606	0.70	1,744 (50.8)	1,350 (39.3)	293 (8.5)	49 (1.4)	0 (0.0)	0.08**	0.13**	0.19**	0.83**	0.17**	0.16**
Allow for unexpected expenses?	0.7	1.697	1.18	572 (16.7)	1,106 (32.2)	801 (23.3)	699 (20.4)	254 (7.4)	0.05*	0.15**	0.21**	0.91**	0.17**	0.19**
Monthly (net) salary	0.8	1.121	0.80	628 (18.3)	1,965 (57.4)	697 (20.3)	62 (1.8)	74 (2.2)	0.15**	0.16**	0.11**	0.81**	0.22**	0.07**
Rights														
Pension	1.3	0.470	0.68	2,162 (63.4)	891 (26.1)	356 (10.4)			0.07**	0.24**	0.08**	0.23**	0.54**	0.05**
Severance pay	2.0	1.239	0.75	634 (18.7)	1,309 (38.7)	1,443 (42.6)			0.04*	0.03	0.01	0.01	0.57**	0.05**
Maternity leave/ parental leave	1.1	0.108	0.42	3,178 (93.0)	109 (3.2)	130 (3.8)			0.07**	0.07**	0.02	0.14**	0.45**	0.01
Unemployment benefit	2.3	0.694	0.87	1,960 (58.1)	487 (14.4)	928 (27.5)			−0.06**	−0.05**	0.02	0.12**	0.68**	0.04*
Exercise rights														
Weekly holidays	1.3	. 567	1.01	2,328 (68.3)	596 (17.5)	204 (6.0)	195 (5.7)	86 (2.5)	−0.01	0.06**	0.16**	0.06**	0.03	0.63**
Sick leave	0.9	0.498	0.97	2,491 (72.8)	487 (14.2)	201 (5.9)	161 (4.7)	83 (2.4)	−0.02	−0.06**	0.26**	0.19**	0.06**	0.69**
Go to doctor	0.9	0.761	1.01	1,845 (53.9)	924 (27.0)	336 (9.8)	271 (7.9)	49 (1.4)	−0.05**	−0.03	0.36**	0.18**	0.06**	0.80**
Holiday	1.2	0.708	0.85	1,624 (47.6)	1,366 (40.0)	269 (7.9)	111 (3.3)	45 (1.3)	0.02	0.01	0.28**	0.13**	0.08**	0.70**
Day(s) off for personal reasons	1.1	1.235	1.19	1,111 (32.5)	1,195 (32.8)	470 (13.8)	462 (13.5)	143 (4.2)	−0.03	−0.06**	0.35**	0.11**	0.04*	0.85**
Day(s) off for family reasons	1.1	1.176	1.18	1,221 (35.7)	1,120 (32.8)	470 (13.8)	462 (13.5)	143 (4.2)	−0.04*	−0.05**	0.36**	0.11**	0.05**	0.85**

Note. ; deviation; *p p< 0.05. **p p< 0.01. *** for results corrected for overlap please see online supplemental material 2.

### Scale descriptive statistics

Table 3 shows means *M*, SD, proportion of missing values, floor and ceiling effects and the subscales’ internal consistency. The mean scores ranged from 0.47 (*temporariness*) to 1.45 (*disempowerment*). The global score (mean of the subscales) of the EPRES-Ge was 0.99. All subscores ranged from 0 to 4, with the global score ranging from 0.00 to 2.80. Although ceiling effects were generally low, floor effects were considerably high for *temporariness* (81.4%), *vulnerability* (18.3%) and *exercise rights* (20.7%).

**Table 3:** Descriptive statistics of the Employment Precariousness Scales of the sample of mothers and their partners at T1

	Items	*M*	*SD*	Missing values (%)*	Observed range	Floor effects (%)†	Ceiling effects (%)‡	Cronbach’s alpha (CI)
Temporariness	2	0.47	1.01	3.04	0–4	81.4	0.5	0.73 (0.71 to 0.75)
Disempowerment	2	1.45	1.0	1.30	0–4	25.4	3.1	0.77 (.75 to 0.78)
Vulnerability	5	0.71	0.68	1.68	0–4	18.3	0.0	0.76 (0.75 to 0.78)
Wages	3	1.24	0.84	1.07	0–4	7.9	0.5	0.79 (0.78 to 0.80)
Rights	4	1.26	0.79	3.13	0–4	10.9	0.6	0.29 (0.25 to 0.33)
Exercise rights	6	0.82	0.78	2.87	0–4	20.7	0.3	0.85 (0.84 to 0.86)
Global score	22	0.99	0.41	9.93	0–2.80	0.3	0.0	0.77 (0.76 to 0.78)

* Proportion in percent of participants with any item missing on the respective subscale.

† Proportion in percent of participants with lowest (0) EPRES-Ge scores.

‡ Proportion in percent of participants with highest^4^ EPRES-Ge scores.

### Exploratory factor analysis

Results of the exploratory factor analysis within the first subsample (n=1,718) are shown in table 4. Scree plot analysis extracted five factors with eigenvalues of >1 (eigenvalues=4.06; 2.35; 1.76; 1.60; 1.20). All items showed the highest loading within their theoretical subscale. All item loadings were above .60 with the exception of the subscale *rights*, where the items exhibited a lower factor loading ranging from 0.45–0.68. The whole model explained 45.08% of the cumulative variance.

**Table 4:** Factor loading of the exploratory factor analysis of the split sample (n=1718)

	Factor				
	1	2	3	4	5
Temporariness					
Duration of contract			0.13	0.90	
Months under temporary contracts previous year				0.87	
Vulnerability					
Afraid to demand better working conditions	0.19	**0.66**	0.14		
Defenseless towards unfair treatment	0.16	**0.76**			
Afraid of being fired for not doing…		**0.58**			
Treated in authoritarian manner	0.19	**0.51**			
Made to feel easily replaceable	0.12	**0.59**			
Wages					
Cover basic needs?		0.13	**0.75**		
Allow for unexpected expenses?		0.16	**0.83**		
Monthly (net) salary			**0.71**	0.15	0.19
Rights					
Pension			0.23		**0.21**
Severance pay					**0.09**
Maternity leave/parental leave					**0.60**
Unemployment benefit			0.11		**0.33**
Exercise rights					
Weekly holidays	**0.57**				
Sick leave	**0.62**	0.17	0.14		
Go to doctor	**0.76**	0.24			
Holiday	**0.63**	0.16			
Day(s) off for personal reasons	**0.67**	0.21			
Day(s) off for family reasons	**0.65**	0.23			

Note. Table showing factor loadings >0.1.

### CFA

Within the CFA, the hypothesised model with five latent factors provided a good fit to the data with CFI=0.981 and RMSEA=0.050 within the second subsample. The chi-square was significant at 846.93 (*df*=161; *p*<0.001), which is common in a large sample.^58^ Item loadings were high with the exception of the items concerning *severance pay* and *unemployment benefit*. Results of the CFA are shown in online supplemental material 3, figure 1. An alternative model with just one latent factor performed worse than the 5-factor model (CFI=0.97; RMSEA=0.06; Chi-square difference=9431.62, *p*<0.001). In addition, when no general factor was specified and items were allowed to correlate freely, the fit was again worse than the fit of the proposed structure (CFI=0.72, RMSEA=0.19).

### Prospective associations of PE during pregnancy with mental health outcomes after birth

Finally, first indications of prospective associations of the EPRES-Ge during pregnancy (T1) with mental health outcomes 8 weeks after birth (T2), namely, the SCL-90-R with its subscales and the EPDS were examined.

Regarding the association of the EPRES and mental health outcomes measured by the SCL-90-R, results are presented in table 5. The scores of the scales ranged from 0 to 34 with mean scores between *M*=1.59–3.80 (see table 1). Using multiple regression analyses with EPRES-Ge as the independent variable, adjusted for age, sex and educational level, the EPRES-Ge score showed statistically significant prospective associations with symptoms of *somatisation, anxiety, obsessive-compulsiveness,* and *hostility* (*β’s*=0.14–.20*, p’s≤0*.001*).* In these analyses, men were presented with fewer symptoms of mental health problems compared with women exposed to the PE. Further multivariate regression analyses were conducted to explore differences by subscale and sex (see table 5). The associations between the EPRES-Ge and the SCL-90-R remained statistically significant when analyses were repeated for either women or men only. For both sexes, only the subscales *vulnerability* and *wages* showed statistically significant positive prospective associations with the SCL-90-R. The only exception was found for the association of the subscale *exercise rights* and *anxiety* (*p=*0.07), which was only found for men (results not shown).

**Table 5:** Effects of Employment Precariousness Scale-Germany score on symptoms of somatisation, anxiety, obsessive-compulsive and hostility (Model 1–4) and symptoms of postpartum depression (Model 5), adjusted for sex, age, and education

		Model 1 (Somatisation)*			Model 2 (Anxiety)*			Model 3 (Obsessive-compulsive)*			Model 4 (Hostility)*			Model 5 (Depression)†		
		β	P	CI	β	P	CI	β	P	CI	β	P	CI	β	P	CI
Overall score	EPRES-Ge	**0.15**	0.00	(0.88, 1.48)	**0.17**	0.00	(0.71, 1.15)	**0.20**	0.00	(1.43, 2.10)	**0.14**	0.00	(0.48, 0.86)	**0.17**	0.00	(4.65, 7.11)
	Sex (female/male)	**−0.18**	0.00	(−1.53, 1.0)	**−0.08**	0.00	(−0.61, −0.21)	**−0.09**	0.00	(−0.99, −0.39)	**−0.12**	0.00	(−0.70, −0.37)	**−0.24**	0.00	(−2.10, 1.52)
	Age (in years)	**0.05**	0.01	(0.01, 0.07)	**0.06**	0.01	(0.01, 0.06)	0.01	0.50	(−0.02, 0.05)	**0.04**	0.05	(0.00, 0.04)	0.01	0.58	(−0.02, 0.04)
	Education (university degree yes/no)	**−0.06**	0.00	(−0.69, −0.17)	**−0.01**	0.67	(−0.23, 0.15)	0.02	0.33	(−0.15, 0.44)	−0.03	0.19	(−0.27, 0.05)	−0.02	0.42	(−0.40, 0.16)
Individual scales	Temporariness	−0.02	0.44	(−0.18, 0.08)	−0.02	0.25	(−0.15, 0.04)	0.02	0.42	(−0.09, 0.20)	−0.00	0.74	(−0.10, 0.07)	−0.02	0.35	(−0.20, 0.07)
	Vulnerability	**0.12**	0.00	(0.40, 0.81)	**−0.02**	0.00	(0.54, 0.85)	**0.20**	0.00	(0.87, 1.33)	**0.13**	0.00	(0.28, 0.54)	**0.16**	0.00	(0.69, 1.13)
	Wages	**0.13**	0.00	(0.36, 0.70)	**0.12**	0.00	(0.22, 0.48)	**0.12**	0.00	(0.36, 0.75)	**0.11**	0.00	(0.17, 0.39)	**0.12**	0.00	(0.36, 0.73)
	Rights	0.01	0.73	(−0.13, 0.20)	0.01	0.759	(−0.10, 0.14)	0.02	0.24	(−0.07, 0.30)	−0.01	0.50	(−0.14, 0.07)	0.02	0.25	(−0.07, 0.28)
	Exercise rights	0.03	0.16	(−0.05, 0.31)	0.00	0.911	(−0.12, 0.14)	0.01	0.60	(−0.15, 0.25)	0.03	0.22	(−0.04, 0.18)	0.02	0.26	(−0.08, 0.30)
	Sex (female/male)	**−0.16**	0.00	(−1.40,−0.86)	**−0.06**	0.003	(−0.50, −0.11)	**−0.07**	0.00	(−0.85, −0.25)	**−0.11**	0.00	(−0.62, −0.29)	**−0.22**	0.00	(−1.95, 1.37)
	Age (in years)	**0.04**	0.04	(0.00, 0.06)	**0.04**	0.045	(0.00, 0.05)	0.00	0.99	(−0.85, −0.25)	0.03	0.20	(−0.03, 0.04)	−0.00	0.88	(−0.04, 0.03)
	Education (university degree yes/no)	−0.01	0.53	(−0.38, 0.19)	**0.04**	0.049	(0.00, 0.42)	**0.06**	0.01	(0.14, 0.78)	0.02	0.45	(−0.11, −0.25)	0.03	0.11	(−0.05, 0.56)

Note. β=standardised beta coefficient; interval; . Significant associations are presented in bold.

* SCL-90-R=Subscale of Symptom-Checklist Revised.

† EPDS=Edinburgh Postnatal Depression Scale.

The EPDS ranged from 0 to 30 with a mean score of 4.91 (*SD=*3.81), representing a fairly healthy sample (see table 1). Using multiple regression analysis with EPRES-Ge as a predictor, adjusted for age, sex, and education, the EPRES-Ge score during pregnancy showed a statistically significant positive association with symptoms of PPD (*β*=0.17, *p*<0.001; table 5).

For both sexes, only the subscales *vulnerability* and *wages* showed statistically significant positive prospective associations with the EPDS. The exception is the *temporariness* subscale, which only showed a marginally significant association with the EPDS for men (*p*=0.07).

## Discussion

### Key findings and summary

According to our findings, the EPRES-Ge is a useful multidimensional instrument for assessing dimensions of PE in Germany for this sample of expectant mothers and their partners, with most participants having a permanent employment contract. The measure yielded a very good response for the respective items with very few missing values and good reliability in almost all subscales. The overall factorial structure was confirmed by exploratory factor analysis with high item-subscale correlations and factor loadings. Construct validity was examined by CFA and the hypothesised five-factor model showed a very good fit to the data. Finally, the total score of the EPRES-Ge predicted several mental health outcomes at 8 weeks postpartum, highlighting the negative impact that PE may have on employees’ mental health.

Concerning *temporariness*, 80% of participants stated having a permanent work contract, yielding a low subscale score. Nevertheless, PE also affects workers with a permanent work contract,^59^ which is reflected in other subscale scores within our sample. As the authors of the EPRES state, the scale *temporariness* might underestimate the EPRES’ association with worker’s health, meaning that employees might score low on this subscale, but the effects of PE in other dimensions could still have an impact on employees’ health.^59^ This was also supported by our findings, making the application of PE measures such as the EPRES in samples of standard employment just as important as in samples of non-standard forms of employment.

A more theoretical explanation may be needed for the low reliability of the *rights* subscale. Cronbach’s alpha can be described as a measure of the reliability and consistency of the sampling instrument. It examines whether all items measure the same underlying construct. German government legislation protecting certain employee rights could explain a low Cronbach’s alpha in this sample. Most employees in Germany are entitled to unemployment benefits as well as pension benefits from the social security system. Therefore, employers are not necessarily expected to provide these benefits themselves. In contrast, most employers are required by law to provide paid parental leave, so the *rights* scale measures different aspects of rights provided by employers and/or the welfare system in the context of the German work environment. However, the association with mental health outcomes, particularly for men, demonstrates the importance of this scale, even though it measures different aspects of the work environment.

Only the subscale *disempowerment* could not be used to distinguish between PE and non-PE in this highly skilled German sample, as participants negotiated working hours and salaries that were in some cases more advantageous for the individual than the conditions imposed by the unions (eg, fewer working hours). Future research should focus on adapting the scale of *disempowerment* to different economic backgrounds. The authors of the EPRES-Se suggested reformulating the items of *disempowermen*t according to the laws in force in each country,^38^ and the authors of the EPRES-E have adapted the scale to assess whether trade unions or work councils are generally represented in the company, whether the employee can exercise control over his or her own working hours and whether regular meetings are held to express the employee’s opinions.^60^

In conclusion, participants in our well-educated sample in Germany scored relatively low on the EPRES as it has been found in other populations of permanent workers in Spain.^36 47^ This was expected due to the unique sample of expectant mothers and fathers who are likely to be in a position to financially support a newborn child.

However, these scores are relatively low compared with other study populations. In the Swedish version of the EPRES, participants scored higher on all dimensions of the EPRES and on the total score (EPRES-Se=1.9). Similarly, participants in Chile also scored higher (EPRES-Ch=1.32), although Chile has a different economic background than Germany, making a comparison more difficult. Recently, an adapted version, the EPRES-E, was validated in Spain during an interview with only 13 proxy indicators^60^ adding the new dimension of *Uncertain working times* to the theoretical structure. A comparison of these EPRES-E scores across 22 European countries, including Germany, has recently been published.^61^ Germany was found to be the third worst-performing country in terms of overall EPRES-E score within 22 European countries. A particularly poor performance was found in the dimension of *wages*,^61^ which was also seen within this sample, where the scale *wages* yielded higher scores in comparison with the other subscales within our population.

There are a few possible limitations that could provide valuable insights into our findings and considerably low subscale scores. First, although the selective sample consisting of expectant parents provides valuable insights into the peripartum period, it is a limitation in terms of the generalisability of the results to other populations. For example, Vives, Benmarhnia^10^ suggested that especially elder employees are more vulnerable to PE and negative general and mental health consequences. As a second limitation, the sample was highly educated (see study protocol; Kress et. al. 2019), making the application of findings to the general population more difficult. Since higher education can lead to more stable and less PE,^10 39^ the transferability from our sample with over 55% of participants holding at least a university degree and 80.0% of participants reporting a permanent contract to the general population might be impaired. Third, the study included only participants with sufficient German language skills and formal employment contracts (full-time, part-time, marginal employment or apprenticeship, employment ban or parental leave), making the sample less representative for working conditions of the foreign-born population and non-standard employment or ‘occasional’ or irregular work. Overall, this could explain the lower EPRES-Ge scores and considerable floor effects found in this investigation of a sample with a high educational background and permanent contracts. To ensure the applicability of the EPRES-Ge in the German work context, we recommend it use in a wider context and in future standardised employee surveys in Germany.

### First prospective associations with mental health outcomes

EPRES-Ge during pregnancy predicted mental health outcomes even after childbirth, when women are typically on maternity leave. This association was evident even after controlling for the major life event of childbirth at T2, 8 weeks after childbirth, and was also true for men who were not protected by the employment ban for women. It should be noted that we did not control for possible changes in employment conditions between T1 and T2, which must be taken into account when interpreting the results. However, in Germany, a pregnant employee is protected against dismissal by the employer under national law. It can therefore be assumed that women’s employment conditions were unlikely to change significantly during pregnancy. In addition, with regard to the period after childbirth, women who have given birth in Germany are not allowed to work for the first 8 weeks after childbirth, while many men continue to work with possible changes in employment conditions. To our knowledge, the present investigation shows the most differentiated results using the EPRES and multiple measures as indicators of mental health, so far. Especially, the scales *vulnerability, wages* and *exercise rights* showed the strongest associations with mental health outcomes. There appears to be a clear gradient pattern between increasing PE and poor mental health.^37 40 41^ Previous research in this area has already suggested that particularly low wages may be a risk factor for pregnant women developing symptoms of PPD after giving birth.^33^ In addition, it has been shown that women in continental welfare states, such as Germany, also tend to score higher on the wage dimension.^21^

This finding is supported by our analysis, where *wages* were more strongly associated with mental health outcomes in women and men than the other EPRES subscales (except *vulnerability*). Low income appears to have a strong impact on family health, even in a high-income country and welfare state such as Germany. The research team using the adapted version of the EPRES-E in countries across Europe confirmed this finding.^61^ Besides Germany being a high-income country, it had the third worst scoring within the subscale *wages.* To add to that, Germany has experienced the strongest growth of in-work poverty in the European Union with close to 10% of workers to be at risk of in-work poverty in 2017.^62^ This suggests that people in Germany might experience a worse ratio of income and living expenses than their European neighbours. Padrosa, Bolíbar^61^ add to the discussion by stating that the rise in low-paid and part-time jobs due to the Hartz reforms might explain the considerable high scores within the German population compared with other European countries.

However, in our analysis, there was no association between *temporariness* and mental health. Wagenaar, Kompier^17^ suggested a possible explanation for this finding in terms of the bi-directional causality of the 'healthy hiring effect', whereby healthy individuals are more likely to be selected for permanent employment.

As mentioned above, research has found that the association between PE and mental health varies by sex. Women were more susceptible to negative consequences of PE, both in general and in relation to mental health.^10 20 63^ However, this difference was not evident in the correlation and regression analyses of the present investigation. Still, further research on possible sex differences is needed, especially since women are over-represented in precarious working conditions such as part-time and marginal employment^64^ and have experienced an increase in low wages during the past years due to economic changes leading to an increase in PE.^63^

Nonetheless, a recent review of atypical employment and mental health in Germany concludes that PE is associated with mental health outcomes; however, the fit with the individual needs of the employer needs to be assessed before this association can be assumed.^65^ But even in a sample with low levels of PE such as ours, the associations found with mental health outcomes make it remarkably important to examine PE in the context of mental health problems. Yet, this study must be interpreted within a wider context. Women’s employment contributes significantly to a country’s economic growth and development. When women participate in the labour force, it increases the total labour force, leading to higher productivity and economic output.^66^ To better understand the context of women’s participation in the labour force, it is crucial to use tools such as the EPRES to assess employment during this period to systematically evaluate the employment conditions that might affect parents during this time. As our results suggest, PE can have a negative impact on mental health even in the presence of relatively favourable working conditions with a low EPRES score in a comparably young population, as was the case in this sample. Given the demographic situation and the importance of mental health, it is important to assess and evaluate PE that may have a negative impact on individual and family health, with consequences for further employment and the upbringing of the next generation of employees, the children.

## Conclusion

The EPRES-Ge proved to be a reliable and valid tool for measuring PE in Germany, with very high acceptability among participants and very few missing values. Although this was the first translation and adaptation, the psychometric properties of five scales were satisfactory. Both exploratory analysis and CFA confirmed the proposed theoretical structure and highlighted the importance of different dimensions of PE. Future research could address the adaptation of *disempowerment* to the German work context. The identified possible prospective associations with mental health outcomes are of great importance for preventive measures targeting risk factors for impaired mental and family health. Therefore, we would like to encourage other researchers and investigators to work with the EPRES-Ge.

## supplementary material

10.1136/bmjopen-2023-077206 online supplemental file 1

## Data Availability

Data are available upon reasonable request.
