# Supplementary material for: Validation of the Employment Precariousness Scale and its associations with mental health outcomes: results from a prospective community-based study of pregnant women and their partners in Dresden, Germany
Source: BMJ Open. 2024 Aug 30;14(8):e077206. doi: 10.1136/bmjopen-2023-077206 (PMC11367369; doi:10.1136/bmjopen-2023-077206)

Supplementary materials:

Validation of the Employment Precariousness Scale (EPRES) and associations with mental health outcomes in a German population of pregnant women and their partners

Marlene Karl[1,2], Andreas Staudt[2,3], Alejandra Vives[4], Marie Kopp[2], Victoria Weise[2], Judith T. Mack[2], Susann Steudte-Schmiedgen[1], Andreas Seidler[2], Susan Garthus-Niegel[2,5,6]

[1] Department of Psychotherapy and Psychosomatic Medicine, Faculty of Medicine, Technische Universität Dresden, Dresden, Germany

[2] Institute and Policlinic of Occupational and Social Medicine, Faculty of Medicine, Technische Universität Dresden, Dresden, Germany

[3] Institute for Community Medicine, Section Methods in Community Medicine, University Medicine Greifswald, Greifswald, Germany

[4] Department of Public Health, Pontificia Universidad Catolica de Chile Escuela de Medicina, Santiago, Chile

[5] Institute for Systems Medicine (ISM) and Faculty of Medicine, Medical School Hamburg, Hamburg, Germany

[6] Department of Childhood and Families, Norwegian Institute of Public Health, Oslo, Norway

Supplements 1

Precarious employment measured by the Employment Precariousness Scale (EPRES-Ge)

Zum Arbeitsvertrag	
Was haben Sie für einen Arbeits-vertrag?	□ Unbefristeter Vertrag
	□ Befristeter Vertrag<br><br>*Beispiele: Vertretungsstelle, Vertrag mit definierter Laufzeit, Werk- oder Dienstvertrag*
	□ Vertrag mit einer Leih- oder Zeitarbeitsfirma
	□ Arbeiten auf eigene Rechnung<br><br>*Selbstständig/ freiberuflich*
	□ Praktikum oder Ausbildungsvertrag

	□ Weiß nicht, Vertragstyp nicht bekannt				
	□ Arbeiten ohne Vertrag				
	□ Sonstiges, und zwar:				
Befristung					
Wie lange sind Sie laut Arbeitsvertrag insgesamt beschäftigt?	□ Ein Jahr oder länger				
	□ Befristet, aber ohne festen Termin				
	□ Zwischen 6 Monaten und weniger als einem Jahr				
	□ Weniger als 6 Monate				
	□ Sonstiges, und zwar:				
Wie lange waren Sie während der letzten 12 Monate insgesamt befristet beschäftigt?<br><br>*Alle befristeten Verträge, Zeit- oder Werkverträge zusammen.*	□ Weniger als 2 Monate				
	□ Zwischen 2 und weniger als 3 Monaten				
	□ Zwischen 3 und weniger als 6 Monaten				
	□ Zwischen 6 und 12 Monaten				
Soziales Klima					
Bezogen auf die Art und Weise, wie Sie bei der Arbeit behandelt werden, wie häufig ...	Immer	Oft	Manchmal	Vereinzelt	Nie
... haben Sie Angst, bessere Arbeits-bedingungen einzufordern?					
... fühlen Sie sich schutzlos gegen-über unfairer Behandlung durch Vorgesetze?					
... fürchten Sie sich vor einer Ent-lassung, wenn Sie nicht das tun, was von Ihnen verlangt wird?					
... werden Sie autoritär behandelt?					
... bekommen Sie zu spüren, dass Sie einfach zu ersetzen sind?					
Verdienst					
	□ Gar nicht				

Ermöglicht Ihnen Ihr Verdienst, Ihre Grundbedürfnisse zu befriedigen?	◻ Etwas
	◻ Ausreichend
	◻ Voll und ganz
Erlaubt Ihnen Ihr Verdienst, größere, unvorhergesehene Ausgaben zu decken?	◻ Nie
	◻ Vereinzelt
	◻ Manchmal
	◻ Oft
	◻ Immer
Wie hoch war Ihr durchschnittlicher monatlicher Nettoverdienst in den letzten 3 Monaten (bzw. vor einem Jahr)?<br><br>*Der Nettoverdienst ist das Arbeitsentgelt, das einem Arbeitnehmer nach allen Abzügen, wie zum Beispiel Einkommenssteuer, Sozialversicherung oder Rentenbeiträge, bleibt.*	◻ Bis 450€
	◻ 451€ bis 850€
	◻ 851€ bis 1.500€
	◻ 1.501€ bis 2.500€
	◻ Mehr als 2.500€
Nutzung betrieblicher Sozialleistungen	

Bezogen auf Ihre Arbeitssituation, sagen Sie bitte, wie oft Sie die folgenden Rechte <u>ohne Probleme</u> wahrnehmen können:	Nie	Vereinzelt	Manchmal	Oft	Immer
Wöchentliche Ruhetage nehmen (z.B. am Wochenende)	◻	◻	◻	◻	◻
Krankheitstage nehmen, wenn nötig	◻	◻	◻	◻	◻
Arzttermine wahrnehmen, wenn nötig	◻	◻	◻	◻	◻
Urlaub nehmen	◻	◻	◻	◻	◻
Freie/n Tag/e nehmen für persönliche Angelegenheiten, wenn nötig	◻	◻	◻	◻	◻
Freie/n Tag/e nehmen für familiäre Angelegenheiten, wenn nötig	◻	◻	◻	◻	◻

Supplements 2

S2. 1 Item-descriptive statistics of the sample of mothers and their partners at T1.

Table S2.1 *Pearson item-subscale correlations corrected for overlap*

Items	Corrected item-scale correlation
**Temporariness**	
Duration of contract	.803
Months under temporary contracts previous year	.803
**Disempowerment**	
Working hours settled	.631
Salary settled	.631
**Vulnerability**	
Afraid to demand better working conditions	.565
Defenseless towards unfair treatment	.659
Afraid of being fired for not doing…	.484
Treated in authoritarian manner	.460
Made to feel easily replaceable	.533
**Wages**	
Cover basic needs?	.689
Allow for unexpected expenses?	.703
Monthly (net) salary	.624
**Rights**	
Pension	.135
Severance pay	.120
Maternity leave/ parental leave	.198
Unemployment benefit	.178
**Exercise rights**	
Weekly holidays	.470
Sick leave	.555
Go to doctor	.694
Holiday	.583
Day(s) off for personal reasons	.755
Day(s) off for family reasons	.750

Supplements 3

S3. Fig. 1 Path diagram for the 5-factor model of employment precariousness (EPRES-Ge)

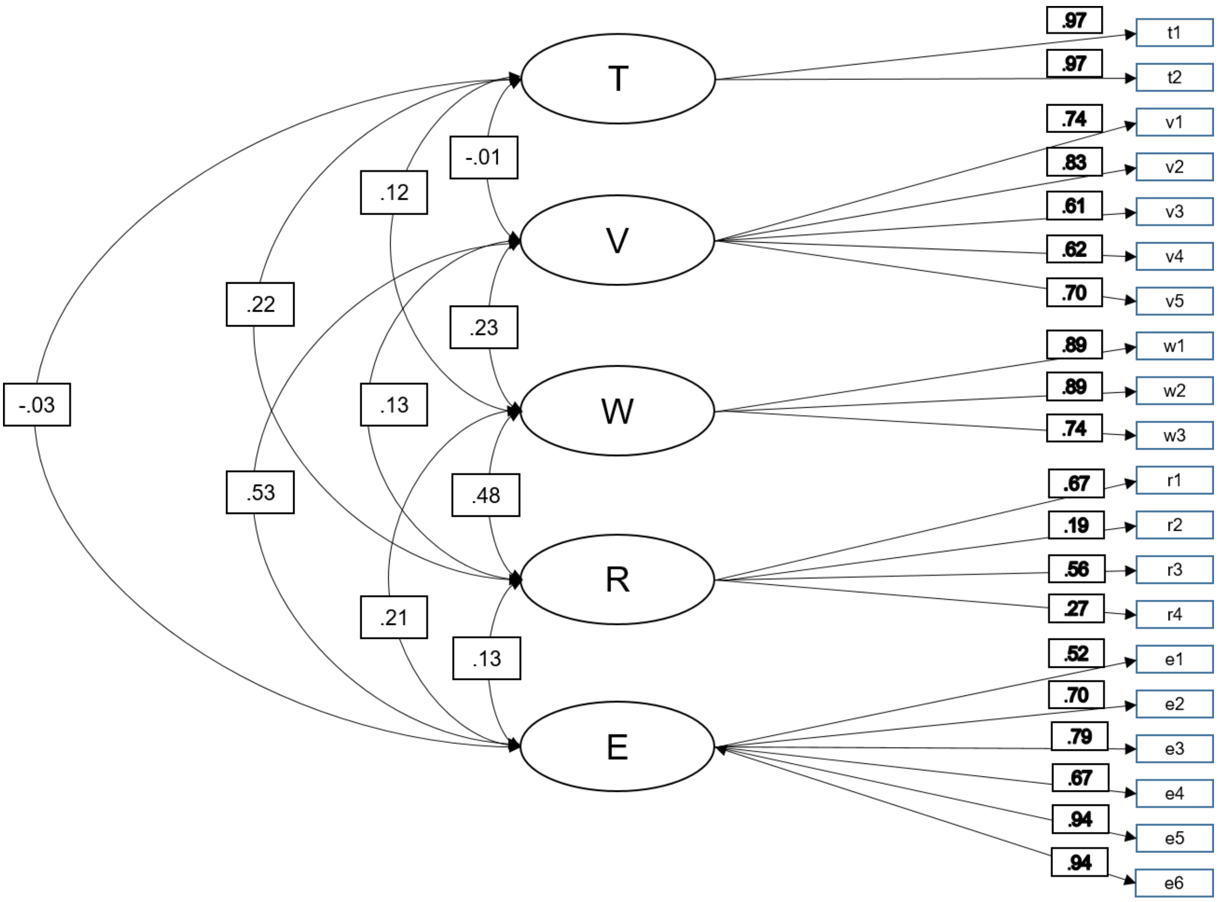

Supplement: online supplemental file 1 [file bmjopen-14-8-s001.pdf]
