# Supplementary material for: Validation of the Employment Precariousness Scale and its associations with mental health outcomes: results from a prospective community-based study of pregnant women and their partners in Dresden, Germany
Source: BMJ Open. 2024 Aug 30;14(8):e077206. doi: 10.1136/bmjopen-2023-077206 (PMC11367369; doi:10.1136/bmjopen-2023-077206)
Supplement: Supplementary file 2 [file bmjopen-2023-077206.reviewer_comments.pdf]

PEER REVIEW HISTORY

BMJ Open publishes all reviews undertaken for accepted manuscripts. Reviewers are asked to complete a checklist review form (**http://bmjopen.bmj.com/site/about/resources/checklist.pdf**) and are provided with free text boxes to elaborate on their assessment. These free text comments are reproduced below.

ARTICLE DETAILS

**TITLE (PROVISIONAL)**	Validation of the Employment Precariousness Scale (EPRES) and associations with mental health outcomes. Results from a prospective community-based study of pregnant women and their partners in Dresden, Germany
**AUTHORS**	Karl, Marlene; Staudt, Andreas; Vives, Alejandra; Kopp, Marie; Weise, Victoria; Mack, Judith T.; Steudte-Schmiedgen, Susann; Seidler, A; Garthus-Niegel, Susan

VERSION 1 – REVIEW

**REVIEWER**	Méndez-Rivero, Fabrizio<br>Universitat Pompeu Fabra
**REVIEW RETURNED**	18-Sep-2023

**GENERAL COMMENTS**	General comments:<br>I suggest considering whether it is not more appropriate to say that it is a validation for Dresden rather than for all of Germany.<br><br>There is an underlying hypothesis that the postpartum period interacts with job insecurity, increasing its impact on women's mental health. This hypothesis should be specified. I suggest complementing the formulation of the main objective with a research hypothesis in this sense.<br><br>Although eight weeks is a short time, there may be changes in employment conditions in that time, how did you consider this in the analysis?<br><br>A section is missing that describes and explains the selection of covariates. For example, what is sex=1?<br><br>Specific comments:<br>Page 2, line 43: It is not necessarily a strength or a weakness.<br>Page 5, line 32: There was a change of reference style<br>Page 4, line 39: Mention the outcomes.<br>Page 6, line 47: What are the items?<br>Page 7 line 7: What are the items?

**REVIEWER**	Demirer, Ibrahim<br>University Hospital Cologne, Institute of Medical Sociology, Health Services Research, and Rehabilitation Science
**REVIEW RETURNED**	18-Sep-2023

**GENERAL COMMENTS**	Your study's objective to validate the EPRES scale with a sample of pregnant women and their partners is commendable. The fact that the EPRES scale has already been validated in several European

	countries using general population or working population samples is a strong foundation.<br><br>Overall your manuscript fails to address the domains of the EPRES in the German context and pathways of precarious employment to mental health. In this context, a heavy emphasis is laid on your study sample context (pregnancy). However, the substantial implications and heavy limitations of it are not presented adequately. Here are my main concerns with your manuscript:<br><br>1) The selection processes between pregnancy and precarious employment are not discussed and contextualized adequatly regarding the aim of your manuscript.<br>2) The selectivity of precarious employment in your study sample, particularly regarding the stability observed at your sample age versus the U-shaped distribution of precarious employment across age.<br>3) The related aspects of voluntary precarious employment, such as its implications for labour market (re-)entry/exit, requires a more comprehensive understanding.<br><br>These factors disable the generalizability of your findings and, consequently, the validation of the EPRES scale within the German context.

VERSION 1 – AUTHOR RESPONSE

Reviewer: 1
Mr. Fabrizio Méndez-Rivero, Universitat Pompeu Fabra
Comments to the Author:
General comments:
I suggest considering whether it is not more appropriate to say that it is a validation for Dresden rather than for all of Germany.
Response: Thank you for this suggestion. Following a request from the editor, we have changed the title to the following and hope that this addresses your point:

"Validation of the Employment Precariousness Scale (EPRES) and associations with men-tal health outcomes. Results from a prospective community-based study of pregnant women and their partners in Dresden, Germany"

There is an underlying hypothesis that the postpartum period interacts with job insecurity, increasing its impact on women's mental health. This hypothesis should be specified. I suggest complementing the formulation of the main objective with a research hypothesis in this sense.
Response: Thank you for pointing this out! We actually did not formulate this specific hypothesis concerning job insecurity and mental health postpartum prior to doing the analysis or research question. We did hypothesize however, that precarious employment with its multiple factors captured by the EPRES would impact the health of employees (men and women). In general, women are more affected by PE and its negative consequences, which was pointed out within the introduction. In addition, we have adapted that part within the introduction and made that more clear by editing the aims of the manuscript (see below).

Introduction:
Against this background, the aim of this study was to (a) translate the revised EPRES-2010 (36) from Spanish into German (EPRES-Ge), (b) to adapt it to the German context, (c) to assess the psychometric properties, and (d) to show prospective associa-tions with mental health outcomes within the peripartum period namely symptoms of depression, somatization, anxiety, obsessive-compulsiveness, and anger/hostility, and further on to explore possible sex differences within this

association. (p.4, lls.25¬–29)

Although eight weeks is a short time, there may be changes in employment conditions in that time, how did you consider this in the analysis?
Reply: Thank you for your comment! The participants in our study completed the first questionnaire during pregnancy (T1), towards the end of the second or beginning of the third trimester. In Germany, a pregnant employee is protected against dismissal by the employer under national law. As the employer also pays part of the maternity benefits, it is very rare for pregnant women to leave their job during pregnancy if they are employed on a permanent or non-permanent contract that covers the period of pregnancy, as was the case for most of our sample. It can therefore be assumed that women's employment conditions did not change significantly during pregnancy. Women are also guaranteed to return to their previous job if they wish to do so after maternity leave. In Germany, women who have given birth are not legally allowed to work during the first 8 weeks after giving birth. Therefore, women were not allowed to work at our time point T2, when we assessed mental health postpartum. However, we do not know whether the partners experienced a change in working conditions. To take account of your comment, we have added this to the section on mental health outcomes in the discussion, which now reads as follows:
EPRES-Ge during pregnancy predicted mental health outcomes even after childbirth, when women are typically on maternity leave. This association was evident even after controlling for the major life event of childbirth at T2, eight weeks after childbirth, and was also true for men who were not protected by the employment ban for women. It should be noted that we did not control for possible changes in employment conditions between T1 and T2, which must be taken into account when interpreting the results. However, in Germany, a pregnant employee is protected against dismissal by the employer under national law. It can therefore be assumed that women's employment conditions were unlikely to change significantly during pregnancy. In addition, with regard to the period after childbirth, women who have given birth in Germany are not allowed to work for the first 8 weeks after childbirth, while many men continue to work with possible changes in employment conditions. (p.16, lls. 33–38)

A section is missing that describes and explains the selection of covariates. For example, what is sex=1?
Response: Thank you for your comment, we fully agree that we should have elaborated more on the selection of covariates in the regression analyses between PE and mental health outcomes. We have added this information and the section now reads as follows:
To investigate prospective associations of PE and mental health outcomes, mul-tivariate regression analyses with the EPRES-Ge score as the independent variable and respective mental health outcomes as dependent variables were conducted. Younger age, anxiety, and low education might be a risk factor for PPD (2) and might be associ-ated with the perception of a less stable/favorable employment as well as female sex, as women typically work in more precarious employment conditions (3). Therefore, we included the covariates age, sex (female/male), and education (university degree yes/no) within our analyses of PE and mental health outcomes. To further explore sex differ-ences within the subscales, regression analyses were repeated separately for women and men. Analyses were performed using SPSS 27 and Mplus version 7.4 (4). (p.6–7, lls. 36–2)

Thanks to your comment, we have also elaborated the interpretation of the confounder sex in the results and discussion and have added the explanation of the confounders in Table 5 (p.15) to allow for a clearer interpretation:

Results:

Finally, first indications of prospective associations of the EPRES-Ge during pregnancy (T1) with mental health outcomes eight weeks after birth (T2), namely the SCL-90-R with its subscales and the EPDS were examined.
Regarding the association of the EPRES and mental health outcomes measured by the SCL-90-R, results are presented in Table 5. The scores of the scales ranged from 0–34 with mean scores between M = 1.59–3.80 (see Table 1). Using multiple regression analyses with EPRES-Ge as the independent variable, adjusted for age, sex, and educa-tion, the EPRES-Ge score showed statistically significant prospective associations with symptoms of somatization, anxiety, obsessive-compulsiveness, and hostility (β's = .14–.20, p's ≤ .001). In these analyses, men presented with fewer

symptoms of mental health problems compared to women when being exposed to the PE. Further multivariate re-gression analyses were conducted to explore differences by subscale and sex (see Table 5). The associations between the EPRES-Ge and the SCL-90-R remained statistically sig-nificant when the analyses were repeated for either women or men only. For both sex-es, only the subscales vulnerability and wages showed statistically significant positive prospective associations with the SCL-90-R. The only exception was found for the asso-ciation of the subscale exercise rights and anxiety (p = .07), which was only found for men (results not shown).
The EPDS ranged from 0–30 with a mean score of M = 4.91 (SD = 3.81), repre-senting a fairly healthy sample (see Table 1). Using multiple regression analysis with EPRES-Ge as a predictor, adjusted for age, sex, and education, the EPRES-Ge score dur-ing pregnancy showed a statistically significant positive association with symptoms of postpartum depression (β = .17, p < .001; Table 5). For both sexes, only the subscales vulnerability and wages showed statistically significant positive prospective associations with the EPDS. The exception is the tempo-rariness subscale, which only showed a marginally significant association with the EPDS for men (p = .07). (p.12–13, lls.13–18)

Specific comments:
Page 2, line 43: It is not necessarily a strength or a weakness.
Response: Thank you for this comment. We have adapted the strengths and limitations sections according to the author guidelines, which now read:
Strengths and limitations:
• This is the first study to translate, validate, and adapt the EPRES, a multidimen-sional instrument for assessing precarious employment in Germany using ex-ploratory and confirmatory factor analyses.
• The validation was done in a large prospective-longitudinal cohort study with a high sample size of 3,455 pregnant women and their partners within the region of Dresden, Germany
• While the selective sample consisting of relatively young women and men ex-pecting a baby provides valuable insights into the peripartum period, it is a limi-tation in terms of the generalizability of the results to other populations.

Page 5, line 32: There was a change of reference style
Response: Thank you for noting! There was a problem with our reference tool. We apologize for that and hope to have corrected all references.

Page 4, line 39: Mention the outcomes.
Response: Thank you for pointing that out. We have added this information.
Against this background, the aim of this study was to (a) translate the revised EPRES-2010 (34) from Spanish into German (EPRES-Ge), (b) to adapt it to the German context, (c) to assess the psychometric properties, and (d) to show prospective associations with mental health outcomes within the peripartum period namely symptoms of depression, somatization, anxiety, obsessive-compulsiveness, and anger/hostility. (p.4. lls.25–29)

Page 6, line 47: What are the items?
Page 7 line 7: What are the items?
Response: Unfortunately, it appears that the line numbers in your version of the manuscript provided by the journal and our line numbers do not match. We assume that you would suggest us to comment further on the EPDS or covariates which we have added:
Furthermore, symptoms of postpartum depression (PPD) were measured by the German version of the Edinburgh Postnatal Depression Scale (EPDS; 48, 49). The EPDS is the most common scale to screen for symptoms of PPD across the perinatal period and has been validated in numerous studies (50). It is a 10-item self-report measure, scored on a four-point scale (0–3), and ranges from 0–30. Higher scores indicate increased levels of PPD. The items of the EPDS refer to feelings of anxiety, guilt or sadness, sleep problems, or thoughts of harming oneself. (p.6, ll.22)
To investigate prospective associations of PE and mental health outcomes, multivariate regression analyses with the EPRES-Ge score as the independent variable and respective mental health outcomes as dependent variables were conducted. Younger age, anxiety, and low education might be a risk factor for PPD (53) and might be associated with the perception of a less stable/favorable employment as well as female sex, as women typically work in more precarious employment conditions (54). Therefore, we included the covariates age, sex (female/male), and education (university degree yes/no) within our analyses of PE and mental health outcomes. (p.6, lls.36–2)

Reviewer: 2
Mr. Ibrahim Demirer, University Hospital Cologne
Comments to the Author:
Your study's objective to validate the EPRES scale with a sample of pregnant women and their partners is commendable. The fact that the EPRES scale has already been validated in several European countries using general population or working population samples is a strong foundation.

Overall your manuscript fails to address the domains of the EPRES in the German context and pathways of precarious employment to mental health. In this context, a heavy emphasis is laid on your study sample context (pregnancy). However, the substantial implications and heavy limitations of it are not presented adequately. Here are my main concerns with your manuscript:

1) The selection processes between pregnancy and precarious employment are not discussed and contextualized adequatly regarding the aim of your manuscript.
Response: Thank you for this complex question. The EPRES questionnaire itself is not pregnancy-related and is suited for all employees. Nevertheless, it is critical to study precarious employment in the peripartum context because of its crucial consequences, especially since this topic has been severely underrepresented in previous research. We do, however, understand that this is a major topic of concern, which is why we have elaborated more on this within the introduction and the discussion.
Introduction:
It has also been hypothesised that PE affects not only general health but also mental health, acting as a stressor on individuals and predisposing them to mental health problems when exposed to it for a prolonged time (5). This association can be found in many European countries, including Germany, where poor self-rated (mental) health was linked with increasing PE (6, 7). However, studying the effects can be quite complex due to various factors such as bidirectional causality, confounding variables, and selection effects (8). In the case of temporary employment, for example, it seems to matter whether workers chose a temporary contract or whether it was the only contract they were offered. Only involuntary temporary employment was associated with nega-tive mental health outcomes (9). Theoretical approaches are still lacking, but initial con-ceptual models link PE and health in the wider context of the political context and its impact on the labour market, together with the consequences of PE such as more haz-ardous working conditions and material deprivation (10). To better understand the underlying contributing mechanisms, recent systematic reviews and a meta-analysis concluded that multidimensional approaches, rather than unidimensional approaches such as insufficient income, yield stronger associations with mental health in terms of effect sizes (9, 11, 12). To add to that, evidence suggests that PE is indeed associated with mental health problems such as psychological dis-tress, depression, anxiety, and mental health symptoms (11, 12), however many longi-tudinal studies on PE and mental health showed limitations within the study design (9).
In particular, women are more exposed to PE (13-15) and psychosocial risk fac-tors than men, resulting in a stronger association between PE and mental health in women in most cases (16). Although different causal pathways between women and men are being discussed in a recent literature review such as the male breadwinner model, where men are responsible to provide for the family while women are respon-sible for care work. (17). New research suggests that not only are mothers at a disad-vantage in the workplace, but that the very fact of age and gender (a woman of childbearing age) results in more PE due to the anticipation of the women having chil-dren, and therefore additional costs to the organisation (18). As then pregnancy itself is a particularly vulnerable time for women, PE must also be considered in the context of peripartum health. It has been highlighted that the pregnancy period, with its many changes and influencing factors, can lead to a greater impact of working conditions on the female worker (19). While employment has been shown to generally have a positive effect on the mental health of mothers (20, 21), some occupational risk factors and working conditions have already been associated with adverse pregnancy outcomes (22-26) with persistent negative effects of PE during pregnancy for the mental health of the mother in the early postpartum period (27, 28). However, research is still sparse on the effect of PE and mental health during the peripartum period. (p.3)
Aim:
Against this background, the aim of this study was to (a) translate the revised EPRES-2010 (1) from Spanish into German (EPRES-Ge), (b) to adapt it to the German context, (c) to assess the psychometric properties, and (d) to show prospective associations with mental health outcomes within the peripartum period namely symptoms of depression, somatization, anxiety, obsessive-

compulsiveness, and anger/hostility. (p.4. lls.25–29)
Discussion:
EPRES-Ge during pregnancy predicted mental health outcomes even after childbirth, when women are typically on maternity leave. This association was evident even after controlling for the major life event of childbirth at T2, eight weeks after childbirth, and was also true for men who were not protected by the employment ban for women. It should be noted that we did not control for possible changes in employment conditions between T1 and T2, which must be taken into account when interpreting the results. However, in Germany, a pregnant employee is protected against dismissal by the em-ployer under national law. It can therefore be assumed that women's employment con-ditions were unlikely to change significantly during pregnancy. In addition, with regard to the period after childbirth, women who have given birth in Germany are not allowed to work for the first 8 weeks after childbirth, while many men continue to work with possible changes in employment conditions. (p.16, lls.22–38)
(…)
But even in a sample with low levels of PE such as ours, the associations found with mental health outcomes make it remarkably important to examine PE in the context of mental health problems. Yet, this study must be interpreted within a wider context. Women's employment contributes significantly to a country's economic growth and development. When women participate in the labor force, it increases the total labor force, leading to higher productivity and economic output (29). To better understand the context of women's participation in the labor force, it is crucial to use tools such as the EPRES to assess employment during this period, in order to systematically evaluate the employment conditions that might affect parents during this time. As our results suggest, PE can have a negative impact on mental health even in the presence of rela-tively favorable working conditions with a low EPRES score in a comparably young population, as was the case in this sample. Given the demographic situation and the importance of mental health, it is important to assess and evaluate PE that may have a negative impact on individual and family health, with consequences for further em-ployment and the upbringing of the next generation of employees, the children. (p.17, lls.30–39)

2) The selectivity of precarious employment in your study sample, particularly regarding the stability observed at your sample age versus the U-shaped distribution of precarious employment across age.
Response: Thank you for this comment! The EPRES has already been validated in other populations, namely in other countries, with a similar age configuration. In Chile 49.1% of the sample were between 25–44 years old (30), and 50.09% were between 26–45 years old in an adapted version of the EPRES that has been validated across Europe (31). In Sweden, 45% of the sample (N=415) for validating the EPRES were between 25–29 years old (32) and 59.1% of the sample between 25-35 years old when applying the EPRES to health outcomes (33).
This is not to justify the particular age range within our sample of 20–56 years with a mean of 31.38 years, but it does put our sample in the context of other validation studies. Because of this validation study, we hope that the EPRES will be used in larger population studies in the future. Thus, we hope to add value to the measurement of precarious employment in Germany.
We did address the issue of this sample within the Strengths and limitations of this study at the beginning of the manuscript, and the limitations within the discussion of the findings, which now read:
Strengths and limitations:
While the selective sample consisting of relatively young women and men expecting a baby provides valuable insights into the peripartum period, it is a limitation in terms of the generalizability of the results to other populations. (p. X lines)
Discussion:

However, a recent review of atypical employment and mental health in Germany concludes that PE is associated with mental health outcomes, but the fit with the indi-vidual needs of the employer needs to be assessed before this association can be as-sumed (34). But even in a sample with low levels of PE such as ours, the associations found with mental health outcomes make it remarkably important to examine PE in the context of mental health problems. Yet, this study must be interpreted within a wider context. Women's employment contributes significantly to a country's economic growth and development. When women participate in the labor force, it increases the total la-bor force, leading to higher productivity and economic output (29). To better under-stand the context of women's participation in the labor force, it is crucial to use tools such as the EPRES to assess employment during this period, in order to systematically evaluate the employment conditions that might affect parents during this time. As our results suggest, PE can have a negative impact on mental health

even in the presence of relatively favorable working conditions with a low EPRES score in a comparably young population, as was the case in this sample. Given the demographic situation and the importance of mental health, it is important to assess and evaluate PE that may have a negative impact on individual and family health, with consequences for further em-ployment and the upbringing of the next generation of employees, the children. (p.17, lls.30–39)

3) The related aspects of voluntary precarious employment, such as its implications for labour market (re-)entry/exit, requires a more comprehensive understanding.
These factors disable the generalizability of your findings and, consequently, the validation of the EPRES scale within the German context.
Response: Thank you for raising this important issue. The items/EPRES were collected during pregnancy (mostly during the 2nd trimester, 29.46 ± 6.37 (8–42)), mainly from first-time parents (96.3%), and related to their work in the last few months. It can therefore be assumed that the responses are representative of this age group (an age group in which the EPRES has already been validated). The EPRES items/responses were not related to pregnancy and did not contain items specifically related to pregnancy. We agree that it may have been difficult to measure/validate the EPRES just before maternity leave, during maternity leave, or after maternity leave, which is why we only considered data from our first time point to assess the validity of the EPRES.
We did not address issues of (re-)entry since we did not measure employment conditions after birth, but, in line with another comment from the other reviewer, we did add the following two statements in our texts and agree that the validation could be extended to an even larger population, which we have added to the limitations that now read:
Discussion:
In order to ensure the applicability of the EPRES-Ge in the German work context, we recommend its use in a wider context and in future standardized employee surveys in Germany. (p.16, lls.26–28)
Discussion
EPRES-Ge during pregnancy predicted mental health outcomes even after childbirth, when women are typically on maternity leave. This association was evident even after controlling for the major life event of childbirth at T2, eight weeks after childbirth, and was also true for men who were not protected by the employment ban for women. . It should be noted that we did not control for possible changes in employment conditions between T1 and T2, which must be taken into account when interpreting the results. However, in Germany, a pregnant employee is protected against dismissal by the em-ployer under national law. It can therefore be assumed that women's employment con-ditions were unlikely to change significantly during pregnancy. In addition, with regard to the period after childbirth, women who have given birth in Germany are not allowed to work for the first 8 weeks after childbirth, while many men continue to work with possible changes in employment conditions. (p.16, lls. 33–38)

References:
1.Vives A, Amable M, Ferrer M, Moncada S, Llorens C, Muntaner C, et al. The Employment Precariousness Scale (EPRES): psychometric properties of a new tool for epidemiological studies among waged and salaried workers. Occupational and environmental medicine. 2010;67(8):548-55.
2.Norhayati M, Hazlina NN, Asrenee A, Emilin WW. Magnitude and risk factors for postpartum symptoms: a literature review. Journal of affective Disorders. 2015;175:34-52.
3.Brady D, Biegert T. The rise of precarious employment in Germany. Precarious work. 31: Emerald Publishing Limited; 2017. p. 245-71.
4.Muthén LK, Muthén BO. . Mplus User’s Guide. Seventh Edition. Los Angeles, CA: Muthén & Muthén. 1998-2012.
5.Benach J, Vives A, Amable M, Vanroelen C, Tarafa G, Muntaner C. Precarious employment: understanding an emerging social determinant of health. Annual review of public health. 2014;35:229-53.
6.Matilla-Santander N, Martín-Sánchez JC, González-Marrón A, Cartanyà-Hueso À, Lidón-Moyano C, Martínez-Sánchez JM. Precarious employment, unemployment and their association with health-related outcomes in 35 European countries: a cross-sectional study. Critical Public Health. 2021;31(4):404-15.
7.Matilla-Santander N, González-Marrón A, Martín-Sánchez JC, Lidón-Moyano C, Cartanyà-Hueso À, Martínez-Sánchez JM. Precarious employment and health-related outcomes in the European Union: a cross-sectional study. Critical Public Health. 2020;30(4):429-40.
8.Wagenaar AF, Kompier MA, Houtman IL, van den Bossche SN, Taris TW. Employment contracts

and health selection: unhealthy employees out and healthy employees in? Journal of Occupational and Environmental Medicine. 2012;54(10):1192-200.
9.Rönnblad T, Grönholm E, Jonsson J, Koranyi I, Orellana C, Kreshpaj B, et al. Precarious employment and mental health: a systematic review and meta-analysis of longitudinal studies. Scandinavian journal of work, environment & health. 2019;45(5):429-43.
10.Benach J, Vives A, Tarafa G, Delclos C, Muntaner C. What should we know about precarious employment and health in 2025? Framing the agenda for the next decade of research. International journal of epidemiology. 2016;45(1):232-8.
11.Jaramillo NR, Trillos CE, Julià M. The measure of precarious employment and its impact on the mental health of workers: A systematic review 2007–2020. Work. 2022(Preprint):1-12.
12.Utzet M, Valero E, Mosquera I, Martin U. Employment precariousness and mental health, understanding a complex reality: a systematic review. International Journal of Occupational Medicine and Environmental Health. 2020;33(5):569-98.
13.Puig-Barrachina V, Vanroelen C, Vives A, Martínez JM, Muntaner C, Levecque K, et al. Measuring employment precariousness in the European Working Conditions Survey: the social distribution in Europe. Work. 2014;49(1):143-61.
14.Menéndez M, Benach J, Muntaner C, Amable M, O'Campo P. Is precarious employment more damaging to women's health than men's? Social science & medicine. 2007;64(4):776-81.
15.Padrosa E, Vanroelen C, Muntaner C, Benach J, Julià M. Precarious employment and mental health across European welfare states: a gender perspective. International Archives of Occupational and Environmental Health. 2022:1-18.
16.Rivero FM, Padrosa E, Utzet M, Benach J, Julià M. Precarious employment, psychosocial risk factors and poor mental health: A cross-sectional mediation analysis. Safety science. 2021;143:105439.
17.Valero E, Martin U, Bacigalupe A, Utzet M. The impact of precarious jobs on mental health: a gender-sensitive literature review. International archives of occupational and environmental health. 2021;94(4):577-89.
18.Peterson Gloor JL, Okimoto TG, King EB. "Maybe baby?" The employment risk of potential parenthood. Journal of Applied Social Psychology. 2022;52(8):623-42.
19.Lee LJ, Symanski E, Lupo PJ, Tinker SC, Razzaghi H, Chan W, et al. Role of maternal occupational physical activity and psychosocial stressors on adverse birth outcomes. Occupational and environmental medicine. 2017;74(3):192-9.
20.Frech A, Damaske S. The relationships between mothers' work pathways and physical and mental health. Journal of Health and Social Behavior. 2012;53(4):396-412.
21.Klumb PL, Lampert T. Women, work, and well-being 1950–2000:: a review and methodological critique. Social science & medicine. 2004;58(6):1007-24.
22.Cai C, Vandermeer B, Khurana R, Nerenberg K, Featherstone R, Sebastianski M, Davenport MH. The impact of occupational shift work and working hours during pregnancy on health outcomes: a systematic review and meta-analysis. American journal of obstetrics and gynecology. 2019;221(6):563-76.
23.Lee BE, Ha M, Park H, Hong YC, Kim Y, Kim YJ, Ha EH. Psychosocial work stress during pregnancy and birthweight. Paediatric and perinatal epidemiology. 2011;25(3):246-54.
24.Meyer JD, O'Campo P, Warren N, Muntaner C. Association of Birthweight With Maternal Trajectories of Effort–Reward Imbalance and Demand–Control Across Pregnancy. Journal of Occupational and Environmental Medicine. 2017;59(2):169-76.
25.Takahata H, Shiraishi M. Association of occupational stress during pregnancy with premature birth and small for gestational age: A systematic review of observational studies. Journal of Japan Academy of Midwifery. 2020;34(1).
26.Corchero-Falcón MdR, Gómez-Salgado J, García-Iglesias JJ, Camacho-Vega JC, Fagundo-Rivera J, Carrasco-González AM. Risk Factors for Working Pregnant Women and Potential Adverse Consequences of Exposure: A Systematic Review. International Journal of Public Health. 2023:35.
27.Karl M, Schaber R, Kress V, Kopp M, Martini J, Weidner K, Garthus-Niegel S. Precarious working conditions and psychosocial work stress act as a risk factor for symptoms of postpartum depression during maternity leave: results from a longitudinal cohort study. BMC Public Health. 2020;20(1):1-11.
28.Schaber R, Karl M, Kopp M, Kress V, Weidner K, Martini J, Garthus-Niegel S. My job, my child, my house: the predictive value of job-and housework-related factors on depressive symptoms during the postpartum period. Journal of Affective Disorders. 2020;272:388-97.
29.Kimmel J. Child care, female employment, and economic growth. Community Development. 2006;37(2):71-85.
30.Vives-Vergara A, González-López F, Solar O, Bernales-Baksai P, González MJ, Benach J.

Precarious employment in Chile: psychometric properties of the Chilean version of Employment Precariousness Scale in private sector workers. Cadernos de saude publica. 2017;33.
31.Padrosa E, Belvis F, Benach J, Julià M. Measuring precarious employment in the European Working Conditions Survey: psychometric properties and construct validity in Spain. Quality & Quantity. 2021;55(2):543-62.
32.Jonsson J, Vives A, Benach J, Kjellberg K, Selander J, Johansson G, Bodin T. Measuring precarious employment in Sweden: translation, adaptation and psychometric properties of the Employment Precariousness Scale (EPRES). BMJ open. 2019;9(9):e029577.
33.Jonsson J, Matilla-Santander N, Kreshpaj B, Johansson G, Kjellberg K, Burström B, et al. Precarious employment and general, mental and physical health in Stockholm, Sweden: a cross-sectional study. Scandinavian journal of public health. 2021;49(2):228-36.
34.Hünefeld L. Atypische Beschäftigung und psychische Gesundheit (Atypical employment and mental health). Arb Soz Umw. 2018;53:32-7.

VERSION 2 – REVIEW

**REVIEWER**	Demirer, Ibrahim<br>University Hospital Cologne, Institute of Medical Sociology, Health Services Research, and Rehabilitation Science
**REVIEW RETURNED**	06-Mar-2024

**GENERAL COMMENTS**	Although I still rate the generalizability of the article as limited, especially with regard to the selection processes on and within the population, I feel that my comments have been sufficiently considered and appreciate the efforts of the authors.

VERSION 2 – AUTHOR RESPONSE

Reviewer: 2
Mr. Ibrahim Demirer, University Hospital Cologne
Comments to the Author:
Although I still rate the generalizability of the article as limited, especially with regard to the selection processes on and within the population, I feel that my comments have been sufficiently considered and appreciate the efforts of the au-thors.

Response: Thank you for reviewing the manuscript again. We believe that the validation and insights within the pregnant population provide added value that outweighs the raised concern of the generalizability. Additionally, we hope to encourage other research-ers in Germany with this publication to use the EPRES measure and provide further in-sight on its use within the German workforce.
